# Transcriptome analysis of perirenal fat from Spanish Assaf suckling lamb carcasses showing different levels of kidney knob and channel fat

**DOI:** 10.3389/fvets.2023.1150996

**Published:** 2023-05-15

**Authors:** María Alonso-García, Aroa Suárez-Vega, Pablo A. S. Fonseca, Héctor Marina, Rocío Pelayo, Javier Mateo, Juan-José Arranz, Beatriz Gutiérrez-Gil

**Affiliations:** ^1^Departemento de Producción Animal, Facultad de Veterinaria, Universidad de León, León, Spain; ^2^Departamento de Higiene y Tecnología de los Alimentos, Facultad de Veterinaria, Universidad de León, León, Spain

**Keywords:** lamb, carcass traits, perirenal fat, fat deposition, gene expression, RNA-seq

## Abstract

**Introduction:**

Suckling lamb meat is highly appreciated in European Mediterranean countries because of its mild flavor and soft texture. In suckling lamb carcasses, perirenal and pelvic fat depots account for a large fraction of carcass fat accumulation, and their proportions are used as an indicator of carcass quality.

**Material and Methods:**

This study aimed to characterize the genetic mechanisms that regulate fat deposition in suckling lambs by evaluating the transcriptomic differences between Spanish Assaf lambs with significantly different proportions of kidney knob and channel fat (KKCF) depots in their carcasses (4 High-KKCF lambs vs. 4 Low-KKCF lambs).

**Results:**

The analyzed fat tissue showed overall dominant expression of white adipose tissue gene markers, although due to the young age of the animals (17–36 days), the expression of some brown adipose tissue gene markers (e.g., *UCP1*, *CIDEA*) was still identified. The transcriptomic comparison between the High-KKCF and Low-KKCF groups revealed a total of 80 differentially expressed genes (DEGs). The enrichment analysis of the 49 DEGs with increased expression levels in the Low-KKCF lambs identified significant terms linked to the biosynthesis of lipids and thermogenesis, which may be related to the higher expression of the *UCP1* gene in this group. In contrast, the enrichment analysis of the 31 DEGs with increased expression in the High-KKCF lambs highlighted angiogenesis as a key biological process supported by the higher expression of some genes, such as *VEGF-A* and *THBS1*, which encode a major angiogenic factor and a large adhesive extracellular matrix glycoprotein, respectively.

**Discussion:**

The increased expression of sestrins, which are negative regulators of the mTOR complex, suggests that the preadipocyte differentiation stage is being inhibited in the High-KKCF group in favor of adipose tissue expansion, in which vasculogenesis is an essential process. All of these results suggest that the fat depots of the High-KKCF animals are in a later stage of development than those of the Low-KKCF lambs. Further genomic studies based on larger sample sizes and complementary analyses, such as the identification of polymorphisms in the DEGs, should be designed to confirm these results and achieve a deeper understanding of the genetic mechanisms underlying fat deposition in suckling lambs.

## 1. Introduction

In some European Mediterranean countries, suckling lamb meat is highly appreciated because of its mild flavor and soft texture (1, 2). In Spain, dairy sheep farms produce both milk and suckling lambs. Lambs produced in this way are characterized by slaughter weights of 9–14 kg and are exclusively milk-fed animals (3). Suckling lamb carcass quality has traditionally been mainly based on weight, fatness, meat and fat color, conformation and carcass composition (4). Several factors, such as breed (5, 6), sex (7, 8), slaughter body weight fatness level (9), and diet, have been reported to influence the quality of lamb carcasses.

Assaf is a semi-fat-tail breed that is a stabilized cross of the East Friesian breed and the fat-tail Awassi breed from Israel (10). Since the introduction of this breed in Spain in 1977 for milk production, the Assaf breed has largely replaced local breeds due to its high milk production potential (11, 12). Regarding lamb meat production, Spanish Assaf lamb carcasses have been shown to be shorter than carcasses of similar weight from other Spanish native breeds, such as Churra and Castellana (13, 14). In relation to fatness traits, and despite the higher fat tail accumulation of Assaf lambs, overall carcass fattening is similar between Manchega and Assaf, although carcasses of Churra and Latxa suckling lambs seem to be fatter (12, 14).

In suckling lamb carcasses, the content of internal adipose deposits, such as perirenal and pelvic fat, also known as kidney knob and channel fat (KKCF), represent a notable proportion of the total fat of lambs during the first weeks of life (15) and are vital for the neonatal animal to maintain its body temperature (16). These depots are used as classical indicators of total carcass fatness (17). Among different carcass parameters, the KKCF percentages in the carcass, either visually assessed or weighed, have been categorized as the best predictors of suckling lamb carcass quality in Spanish breeds (4, 18). The tissue composition of the leg, loin, or shoulder can also be used for carcass quality prediction; however, all abovementioned traits are difficult to measure during routine production (4). Thus, at the commercial level in Spain, it is common practice to classify carcasses in which the kidneys are completely covered by fat as high-quality suckling lamb carcasses because there is a positive correlation between perirenal fat and global carcass fatness [Commission Regulation (EC) No 2107/1999]. In addition, due to its ability to produce adipokines, perirenal fat is considered not only a deposition tissue but also an important metabolically active tissue (19, 20).

Studies based on RNA-seq technology provide a view of the extent and complexity of eukaryotic transcriptomes, which are essential for revealing the molecular constituents of cells and tissues and for understanding development and disease (21). RNA-seq also provides quantitative measures of gene expression levels and allows the discovery of novel transcribed regions (22). In livestock, the study of transcriptomes has been used to identify the expression of genes in several tissues related to productive traits, such as the mammary gland (23) or intramuscular fat (24) in dairy cattle, adipose tissue in pigs (25, 26), and fat depots in suckling lambs (27).

In relation to fatness traits associated with carcass quality, some RNA-seq studies have analyzed the transcriptome profiles associated with different fat depot deposition patterns by comparing different breeds of the same livestock species, such as cattle (28) or pigs (29). In sheep, the majority of RNA-Seq studies aimed at understanding fat metabolism have focused on the fat tail transcriptome in adult sheep (30–35). In suckling lambs, considering the interest in internal adipose deposits as indicators of carcass fatness (17) and carcass quality (4), our group has previously compared the perirenal fat transcriptome of Churra and Assaf suckling lambs, which show different carcass fatness levels, quantified based on the KKCF indicator trait (27). However, no research has yet been performed to compare suckling lambs of the same breed with different carcass KKCF contents.

In the interest of gaining a deeper understanding of the functional genomic mechanisms associated with carcass quality in suckling lambs, this study aimed to reveal the metabolic processes underlying the control of fatness levels in Assaf suckling lambs. To this end, we performed a comparative analysis between RNA-seq datasets from the perirenal fat of Spanish Assaf suckling lambs showing different KKCF carcass contents. The results presented here provide additional information to better understand the processes underlying fat deposition in sheep. Further studies must confirm whether these processes are common to other ovine breeds or whether breed-specific mechanisms may influence the fatness levels of suckling lamb carcasses.

## 2. Materials and methods

### 2.1. Animals and phenotypes

The 17 Spanish Assaf suckling lambs initially considered in this work were reared at the Instituto de Ganadería de Montaña (IGM; Grulleros, León, Castilla y León, Spain). All of them were males born in the same lambing season (January–February 2020) from Spanish Assaf dairy ewes reared under the same management and diet conditions. The average birth weight of the considered lambs was 4.65 ± 1.07 kg. After birth, the lambs were kept with their dams for 4–8 h to consume the colostrum. Then, they were fed artificial milk until slaughter to avoid the influence of maternal effects. The lambs were held in lamb pens and fed using Ovilac 60 (Calfvet®) milk replacer powder *ad libitum* using a milk replacer machine. The lambs were routinely weighed, and all of them were weighed 24 h before slaughter, which was performed at a local slaughterhouse at the market weight, with an average slaughter weight of 11.21 ± 0.74 kg. The average age at sacrifice was 23.71 ± 4.93 days, ranging between 17 and 36 days. The management, transport, and sacrifice of the animals were in accordance with Spanish and EU legislation [Spanish Laws 32/2007, 6/2013, and RD 37/2014; Council Regulation (EC) 199/2009]. According to the Research Ethics Committee of the University of León, formal ethical approval was not necessary for this case, as the studied lambs were not subjected to any experimental protocols.

At slaughter, phenotypic characterization of the carcasses of the 17 considered lambs was performed. Briefly, at 24 h post-mortem, the lambs’ carcasses were split into two halves at the slaughterhouse. The right halves were weighed and used for carcass quality analysis. Some of the visceral organs and tissues, including the perirenal fat, were removed, and the half carcass was weighed again and jointed (36). Tissue samples from perirenal fat were collected from all sacrificed lambs. The tissue was preserved in an RNA-stabilization solution (Ambion RNAlater; Life Technologies) and stored at 4°C for 24 h. Thereafter, the RNA-stabilization solution was removed, and the samples were frozen at −80°C until RNA extraction.

### 2.2. Selection of animals for the RNA-seq study

To perform a comparison of the transcriptomes of lambs with higher and lower internal fat depot contents, we ranked the 17 studied animals based specifically on the percentage of KKCF in the half carcass. The average value for this phenotype, which showed a normal distribution, was 2.23 ± 0.64. Considering the ranked list of animals based on KKCF phenotype values, we selected the four lambs showing the highest values and the four lambs with the lowest values for this trait, which were defined as the High-KKCF group (*n* = 4; average: 3.23 ± 0.47) and the Low-KKCF group (*n* = 4; 1.65 ± 0.16), respectively (see details in Supplementary Table S1). The contrasting KKCF levels between these two groups of animals were confirmed with a paired *t*-test analysis (value of *p* = 0.0023), and the corresponding perirenal fat RNA samples from these eight animals were selected for the subsequent transcriptomic analysis.

For the selected samples, RNA extraction was performed using the miRNeasy Mini Kit (Qiagen) modified for use in adipose tissue. The RNA integrity value of the samples was measured using an Agilent 2100 Bioanalyzer device (Agilent Technologies), obtaining values above 7.2 for all samples. Once RNA was obtained, cDNA library construction and sequencing were conducted at Novogene in Cambridge (United Kingdom) using the UltraTM RNA Library Prep Kit (NEBNext®). Using a minimum of 0.8 μg of RNA per sample, eight stranded paired-end libraries (four for the High-KKCF group and four for the Low-KKCF group) were prepared. The cDNA libraries were sequenced to a minimum depth of 30 million paired-end reads on a NovaSeq 6000 system (Illumina), generating stranded paired-end reads of 150 bp, according to the manufacturer’s instructions. The raw datasets derived from sequencing are available at the BioStudies repository,^1^ with accession number E-MTAB-11839.

### 2.3. Quality control, alignment, and transcriptomic gene expression profile

First, the FastQC program (v0.11.8) (37) was used to assess the quality of the raw RNA sequences. Later, to map only those sequences with good quality, all sequences were filtered and trimmed by Trimmomatic (v.0.39) (38) using the following parameters: Leading: 5, Trailing: 5, Slidingwindow: 4:20, and Minlen: 36. Then, we used STAR (v.2.7.6a) (39) to map the sequence reads against the reference ovine genome assembly available in Ensembl (Oar_Ram_v1.0_r101). The genome index was generated using the --sjdbOverhang 149 option because the sequences had an average length of 150 bp. The following mapping options were used during the alignment: --outFilterType BySJout --outSAMtype BAM Unsorted SortedByCoordinate --quantMode TranscriptomeSAM --outWigType bedGraph --outWigStrand Stranded. To quantify the expression of each gene, we employed HTSeq-counts (v.0.6.1p1) (40) using the intersection-strict mode. Then, the DESeq2 R package (v.1.32.0) (41) was used to perform the normalization of the counts to fragments per kilobase of exon per million fragments mapped (FPKM) units in each of the groups selected. In this work, genes were considered expressed in the perirenal fat transcriptome when their counts reached 0.01 FPKM. The genes showing expression levels greater than 150 FPKM in the two groups were considered core representative genes (CRGs) of the perirenal fat depots of Spanish Assaf suckling lambs. Together with the CRGs, we also analyzed the FPKM expression values of a list of genetic markers of different fat tissues defined based on the literature (42–44).

To further evaluate the functional mechanisms associated with the list of core genes, a Gene Ontology (GO) enrichment analysis, considering the three established GO categories (Molecular Function, Biological Process, and Cellular Component), was performed with the clusterProfiler R package (45). For these enrichment analyses, the org.Hs.eg.db R package (46) was used as an annotation reference. The false discovery rate (FDR) method was used for multiple testing correction (pAdjustMethod), with a minimum cut-off value (qvaleCutoff) of 0.05.

### 2.4. Differential gene expression and enrichment analysis

Before attempting the differential expression (DE) analysis between the transcriptome datasets of the High-KKCF and Low-KKCF groups, we assessed the variability of the lambs selected for the RNA-Seq study for important factors that could influence carcass fatness levels, such as the type of lambing (double or simple), the lamb’s age (in days), and the birth weight (kg) and slaughter weight (kg; Supplementary Table S1). To test the potential associations of any of the mentioned factors with the KKCF variable, individual beta regression analyses were performed for each factor with the betareg R package (47). These analyses showed that the perirenal and cavitary fatness content, or KKCF variable under study, was significantly dependent on the lamb’s age (value of *p* = 0.028), whereas no significant associations were identified for the other tested factors. Based on this, age was included as a categorical variable in the later DE analysis. The categorical variable “age” was defined based on the median age at slaughter of the eight lambs included in the transcriptomic study (Me = 24.5 days); hence, the samples were divided into two groups according to their age at slaughter: “Young” (*n* = 4, 20.5 ± 2.38 days) and “Old” (*n* = 4, 30.25 ± 4.27 days; see Supplementary Table S2).

Later, using DESeq2 (41), we performed DE analysis between the High-KKCF and Low-KKCF groups, including the age category in the model. DESeq2 normalizes raw gene counts by correcting the data based on library size and RNA composition bias and assessing dispersion through biological replicates. For this analysis, genes with an expression of fewer than 10 counts in at least half of the samples were eliminated. Hence, fold changes and their associated Wald test value of *p* values were obtained by performing pairwise comparisons between the High-KKCF and Low-KKCF groups and between the young and old animals for each gene and based on a negative binomial model. After the differential expression analyses, two lists of genes were obtained with an associated value of *p* adjusted (*p*_adj_) for multiple testing using the false discovery range method proposed by Benjamini and Hochberg (48) (FDR-B&H), one list for each of the comparisons tested in the model (age and perirenal fatness). Only those DEGs with a *p*_adj_ (FDR-B&H) < 0.05 were considered differentially expressed.

The functional mechanisms associated with the identified DEGs were evaluated by performing GO enrichment analyses with the clusterProfiler R package (45) using the previously indicated parameters. The cnetplot function of this package was used to visualize the network of linkages among genes and biological concepts. The options used to construct the network were showCategory = 5 (number of enriched terms to display), categorySize = “*p*_value_” [sizes of nodes by the enrichment *p* value using the format −log10 (value of *p*)], and foldchange = genelist (numeric vector with the fold changes of the genes). Moreover, we used the pairwise_termsim function to calculate the similarity between nodes using the Jaccard similarity coefficient. Then, we used treeplot to visualize enriched terms as a tree, where gene sets with high similarity are clustered together.

## 3. Results

### 3.1. Sequencing, alignment, and gene expression levels

For the perirenal fat transcriptomes of the eight analyzed suckling lambs, approximately 30 million (30,155,920 ± 1,586,425) paired-end reads were generated per sample. Among those reads, 27 million (26,969,992 ± 1,718,628) paired-end reads per sample aligned uniquely to the ovine reference genome (Oar_Ram_v.1.0_r101), which corresponded to ~89% of the total reads.

**Figure 1:**
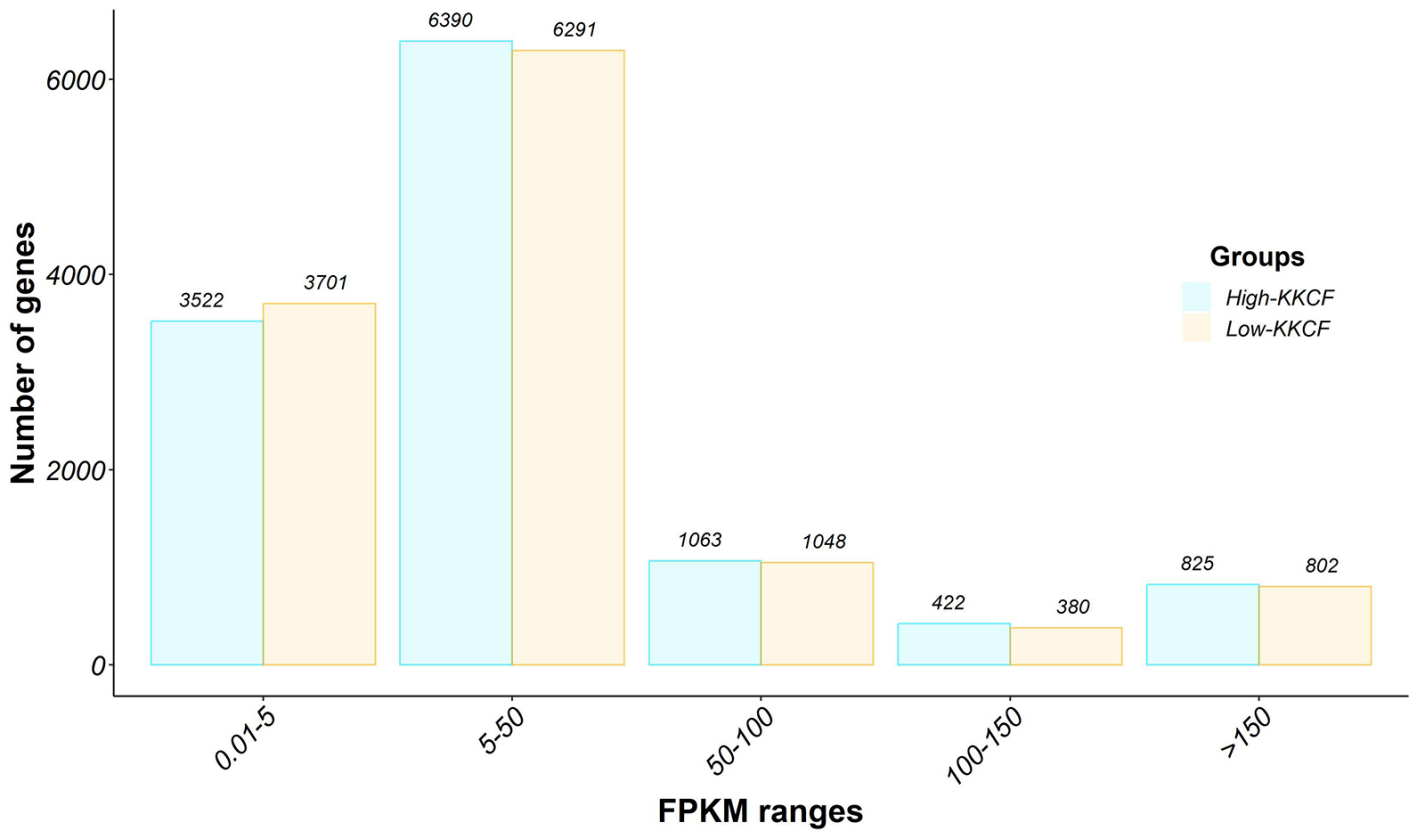
Gene expression distribution of the perirenal fat transcriptomes of the two groups of Assaf suckling lams considered in this study, High-KKCF (in blue) and Low-KKCF (in orange). The bar graph displays the expression intensity distribution of the transcripts identified in the datasets analyzed. The number of genes is represented on the *Y*-axis, whereas the *X*-axis represents the different expression level groups considered for the classification, in fragments per kilobase per million mapped (FPKMs): 0.01–5, 5–50, 50–100, 100–150, and more than 150 FPKMs. The number of genes within each category and group is indicated at the top of each bar.

Out of the 20,506 annotated genes in the Oar_rambouillet_v1.0 sheep assembly, 17,061 genes were classified as expressed (> 0.01 FPKM) in the analyzed perirenal fat transcriptome samples. The distribution of these genes depending on their expression levels is shown in Figure 1. According to an expression level > 150 FPKM, we identified 825 genes and 802 genes in the High-KKCF and Low-PG groups, respectively. From these two lists of genes, 733 genes were shared between the two compared groups. These genes, which represented ~90% of the most expressed genes, were considered the core representative genes (CRGs) of the perirenal adipose transcriptome tissue of Spanish Assaf suckling lambs. Notably, in the list of core genes defined in this study provided in Supplementary Table S3, 137 were annotated as novel Ensembl genes in the considered ovine reference genome (Oar_Ram_v1.0_r101) and did not show an orthologue in humans or cattle. Therefore, 596 annotated genes from Supplementary Table S3 were considered CRGs in subsequent analyses. Based on the GO enrichment analyses performed for this list of annotated CRGs, the most significantly enriched terms identified for the different categories were “*electron transfer activity*” (molecular function), “*aerobic respiration*” (biological process), and “*inner mitochondrial membrane protein complex*” (cellular component). Additional details of this enrichment analysis performed with clusterProfiler, including the genes supporting the significantly enriched terms identified, are provided in Supplementary Table S4.

**Figure 2:**
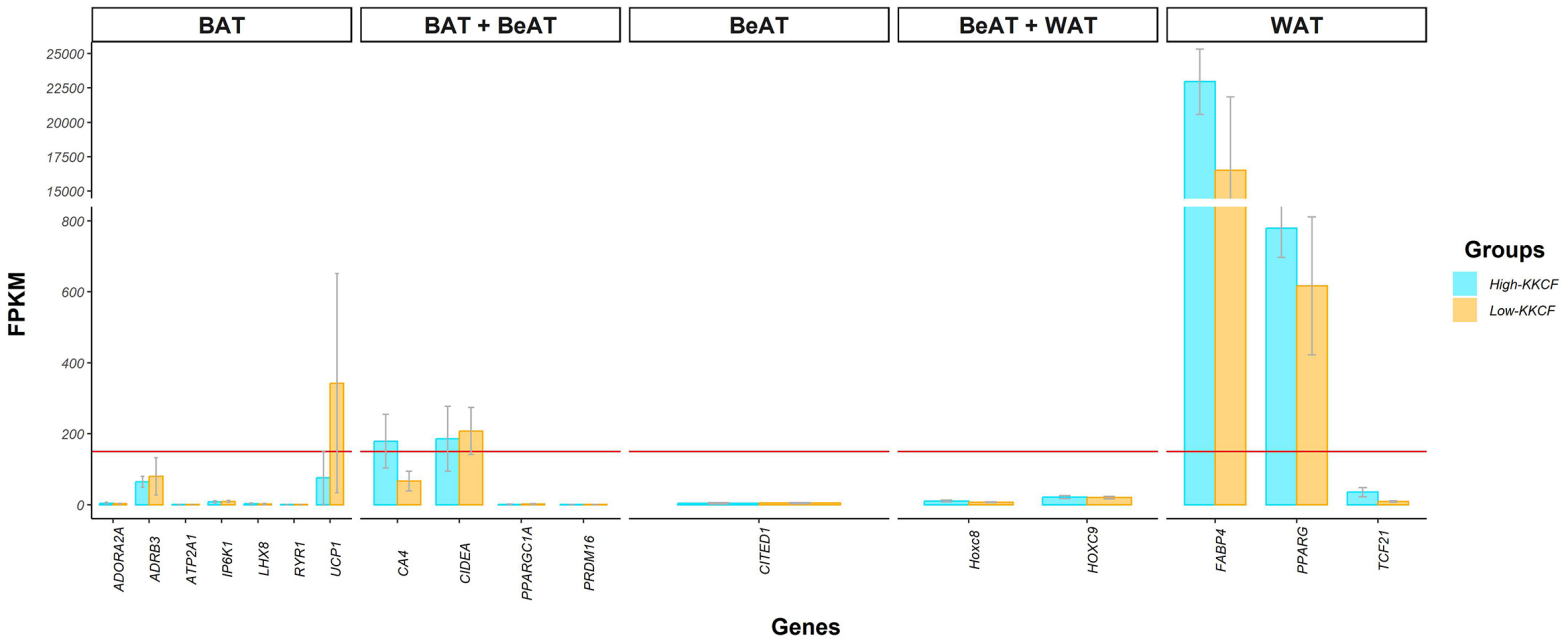
Bar chart showing the gene expression levels, in FPKMs, of different genetic markers reported in the literature for the different types of adipose tissue; brown (BAT), beige (BeAT), and white (WAT) adipose tissue, for the two groups of perirenal fat transcriptomes of suckling lambs analyzed: High-KKCF group (blue) and Low-KKCF group (orange). Each of the bars indicates the mean expression of the genes on the *X*-axis and the gray lines of each bar indicate the standard deviation of expression for each gene in the two groups of samples compared. The horizontal red line indicates the expression threshold level of >150 FPKMs considered to define the genes belonging to the core representative genes (CRG) of the suckling lamb fat transcriptome.

Among the top 10 most highly expressed genes in each group, as shown in Table 1, eight were shared between the two groups. Two of these eight genes were classified as novel genes in the Ensembl database for sheep. According to the external NCBI database, these novel genes, *ENSOARG00020020554* and *ENSOARG00020011816*, were related to eukaryotic translation elongation factor 1 alpha (*EEF1A1*; Acc: 100101228) and ribosomal protein lateral stalk subunit P2 (*RPLP2*, Acc: 101103196), respectively. In addition to Table 1, the gene expression results for some genes are also represented in Figure 2, where the average expression levels of some fat gene markers are presented. The list of gene markers was defined based on the literature (42–44) and included the fat gene markers for the different types of adipose tissue (brown, beige, and white; BAT, BeAT, and WAT, respectively).

**Table 1:** Ten most expressed genes in each of the groups studied when comparing the level of kidney knob and fat carcass (KKCF), High-KKCF and Low-KKCF groups.

Gene ID	FPKM Low-KKCF	Gene ID	FPKM High-KKCF
FABP^*^	16502.17	FABP4^*^	22951.41
*COX1*	9861.54	FTH1^*^	9910.41
TPT1^*^	8545.10	TPT1^*^	7801.98
FTH1^*^	8532.61	ENSOARG00020020554^*^	6824.34
RPLP1^*^	6469.7	RPLP1^*^	6655.42
RPS2^*^	5943.79	*ADIRF*	6558.55
ENSOARG00020020554^*^	5800.31	RPL37^*^	5992.34
RPL37^*^	4993.94	RPS2^*^	5524.44
ENSOARG00020011816^*^	4449.97	*VIM*	5430.51
*ENSOARG00020022219*	4439.93	ENSOARG00020011816^*^	5411.88

Gene expression is measured in fragments per kilobase of transcript per million fragments mapped (FPKM). The genes marked with an asterisk are shared between the two lists.

### 3.2. Differentially expressed genes in the perirenal fat transcriptome

The DE analysis performed between the High-KKCF and Low-KKCF groups considering age as a categorical variable (Young and Old groups), based on the identified association between the KKCF variable and the age in days at slaughter, identified a list of 80 DEGs in the comparison of High-KKCF vs. Low-KKCF.

Among the 80 DEGs identified in the fatness comparison, 49 showed higher expression in the Low-KKCF group, and 31 showed significantly higher expression in the High-KKCF group (Supplementary Table S5). For the sake of simplicity, genes with higher expression in the Low-KKCF group in the abovementioned analysis are referred to as LowKKCF-DEGs. Likewise, genes overexpressed in the High-KKCF group will henceforth be referred to as HighKKCF-DEGs. The heatmap based on the expression levels of the 80 identified DEGs, which is provided in Supplementary Figure S1, shows a clear distinction between the expression profiles of the two contrasted groups (darker colors indicate higher expression in normalized counts).

**Figure 3:**
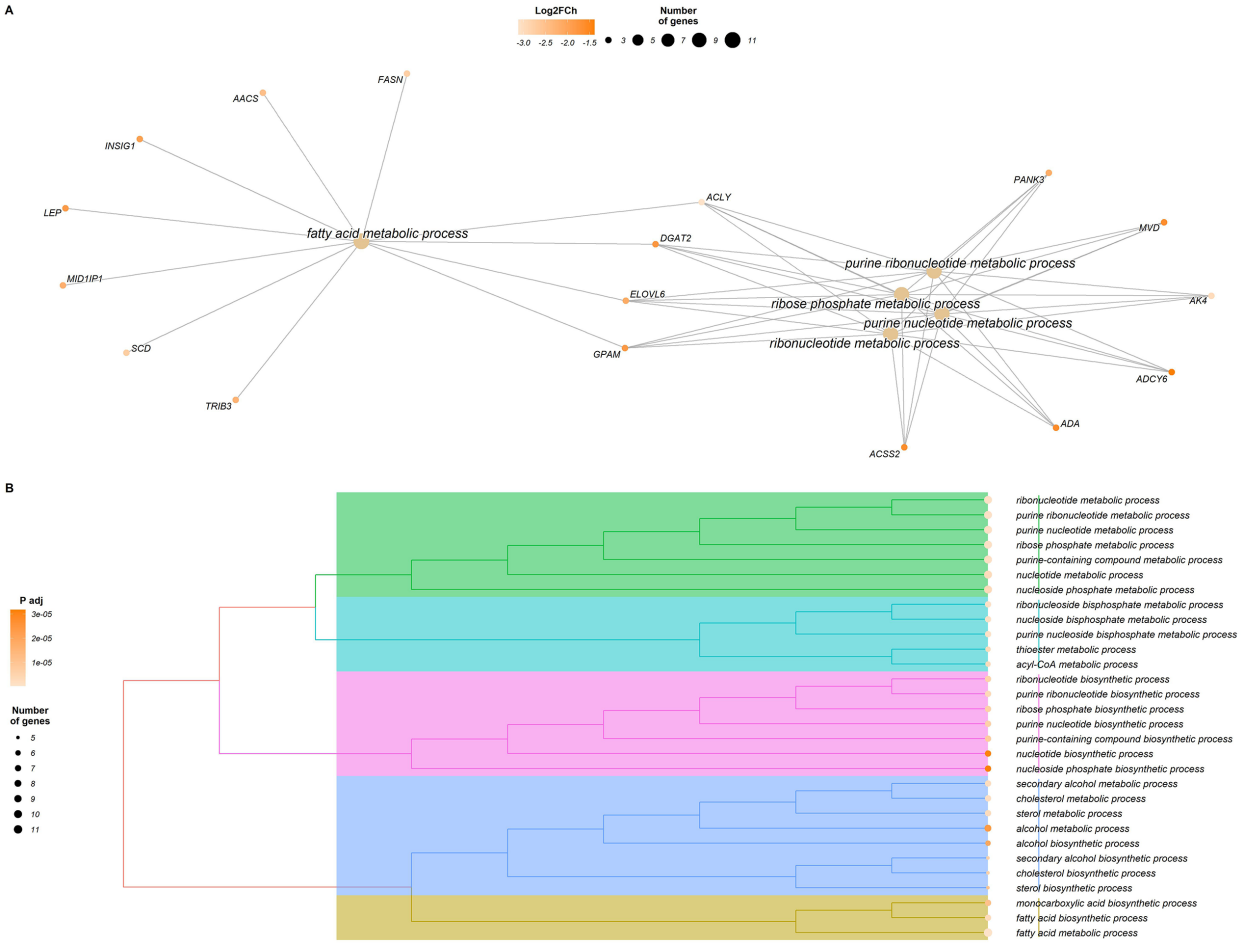
Graphical representation of the results of the enrichment analysis performed with clusterProfiler for the differentially expressed genes (DEGs) in the Low-KKCF group (LowKKCF-DEGs). **(A)** Network of enriched terms and LowKKCF-DEGs that are related to these terms. The orange color scale for the genes indicates their corresponding Log2FoldChange value. **(B)** Dendrogram of the significant enriched terms identified for the LowKKCF-DEGs showing their clustering according to their biological relationship. The size of the circle associated to each term is proportional to the number of genes supporting it. The color intensity of the circle depends on the significance level (*p* value adjusted according to the FDR correction for multiple testing).

For the 41 genes identified as LowKKCF-DEGs (29 annotated genes), we identified 149 significantly enriched GO terms (*p*_adj_-valueFDR <0.05, Supplementary Table S6). Among these terms, 137 belonged to the biological process category, seven belonged to the molecular process category, and the rest (five) were included in the cellular component category. The graphical representation of the LowKKCF-DEGs supporting these enriched terms can be seen in Figure 3. Most of the enriched terms identified in the GO biological process (BP) category were related to lipid metabolism process (Supplementary Table S6), such as cholesterol biosynthesis (“*cholesterol metabolic process*,” “*secondary alcohol metabolic process*,” “*cholesterol biosynthetic process*,” etc.), metabolic processes involving acyl and acetyl-CoA (acyl-CoA and acetyl-CoA metabolic process, etc.) and fatty acid biosynthesis (“*monocarboxylic acid biosynthetic process*,” “*fatty acid biosynthetic process*,” “*fatty acid metabolic process*,” etc.). In addition, for the LowKKCF-DEGs, most of the enriched terms in the GO cellular component (CC) category were related to the endoplasmic reticulum (“*integral and intrinsic component of endoplasmic reticulum membrane*,” integral and intrinsic component of organelle membrane), while one other term was associated with mitochondrial function (“*mitochondrial inner membrane*”); these results were closely related to the terms enriched in the GO molecular function (MF) category, most of which were related to acyltransferase activity (Supplementary Table S6).

**Figure 4:**
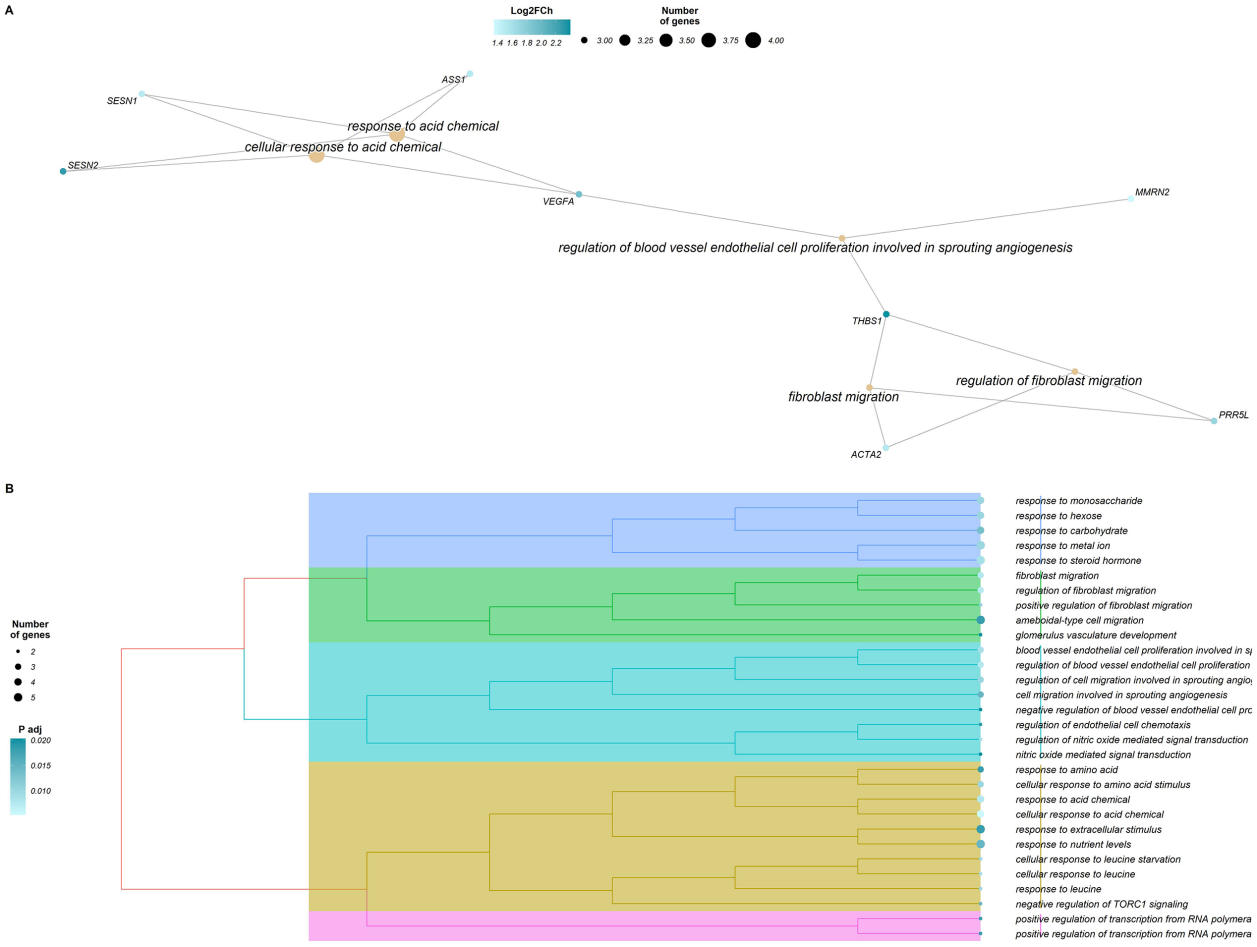
Graphical representation of the results of the enrichment analysis performed with clusterProfiler for the differentially expressed genes (DEGs) in the High-KKCF group (HighKKCF-DEGs). **(A)** Network of enriched terms and HighKKCF-DEGs that are related to these terms. The orange color scale for the genes indicates their corresponding Log_2_FoldChange value. **(B)** Dendrogram of the significant enriched terms identified for the HighKKCF-DEGs showing their clustering according to their biological relationship. The size of the circle associated to each term is proportional to the number of genes supporting it. The color intensity of the circle depends on the significance level (*p* value adjusted according to the FDR correction for multiple testing).

The enrichment analysis performed for the 36 HighKKCF-DEGs (27 annotated) identified a total of 76 significantly enriched GO terms (*p*_adj_-valueFDR < 0.05, Supplementary Table S7). All of these terms belonged to the BP category and were related to different types of cellular responses (“*cellular response to leucine*,” “*cellular response to acid chemical*,” “*cellular response to leucine starvation*,” “*response to acid chemical*,” and “*response to steroid hormone*,” among others), renal development (“*renal system*,” kidney and nephron development, and kidney and glomerulus system vasculature development) and cellular chemotaxis related to angiogenesis (“*regulation of endothelial cell chemotaxis*,” “*cell migration involved in sprouting angiogenesis*,” and more). A graphical representation of these enriched terms and the associated genes is given in Figure 4.

In the list of six DEGs identified in the comparison between the Young and Old categories, three genes showed higher expression in the Young group, and three showed significantly higher expression in the Old group (Supplementary Table S8). Due to the low number of DEGs in the comparison between the Young and Old groups (three and three DEGs, respectively), an enrichment analysis was not performed.

## 4. Discussion

Some carcass parameters, such as kidney knob and channel fatness scores or loin and leg tissue compositions, have been proven to be useful predictors of the composition and quality of suckling lamb carcasses (4). However, the molecular mechanisms of fat metabolism are complex, and characterizing the basis of fat deposition in livestock species may be relevant for maximizing the advantages that can be achieved through genetic selection. In general, previous studies have investigated the transcriptomic profiles of fat tissue between different sheep breeds (27, 33, 35, 49). However, there are no available studies comparing suckling lambs of the same breed with differences in fat deposition.

Research on different mammalian species has revealed dynamic features of fat tissue. Depending on the cellular lineage from which they originate, there are at least three different categories of adipocytes: brown, white, and brite or beige adipocytes (50, 51). In both large and small mammals, brown adipose tissue (BAT) depots are very important in the early stage after birth (52, 53). In fetal sheep, beginning in mid-gestation, multilocular adipocytes, characteristic of BAT tissue, are clearly visible (50). Then, close to term, the fat deposits increase in size, and a mixture of brown and white adipocytes is observed (54). After this time point, coinciding with the time of birth, brown adipocytes gradually disappear until all fat deposits consist of white adipose tissue (WAT). Beige or brite adipocytes, found in beige adipose tissue (BeAT), are very similar to brown adipocytes; however, they come from different cell lines, sharing common cell precursors with white adipocytes (55, 56). Focusing on the BAT to WAT transition, it is not clear how long the period of gradual BAT disappearance lasts. Basse et al. (57) reported that this transition occurs before the first 15 days of life. In contrast, based on comparing the perirenal fat of Churra and Spanish Assaf suckling lambs, Suárez-Vega et al. (27) reported that BAT was still present in some animals at approximately 20 days of age, mainly in Assaf lambs, which were younger at slaughter. These observations agree with the findings of Clarke et al. (58), who reported a large increase in lipid deposition from 7- to 30-day-old ewe lambs, associated with a decline in the amount and activity of UCP to basal values in the ewe lambs. These authors suggested that the precise time scale of this transition process could be regulated in part by the lamb’s body temperature, which determines whether adipose tissue is required for heat production (i.e., BAT) or as an endogenous energy source (i.e., WAT). In this context, considering the expression levels of some of the most representative markers of BAT, BeAT and WAT (according to various authors) in the KKCF suckling lamb samples analyzed here shown in Figure 2, we can see that although WAT markers show the highest expression levels, the expression of some BAT gene markers, such as *UCP1* and *CIDEA*, can still be identified. Hence, to properly interpret the results of further analyses, it is important to consider that the perirenal fat samples of suckling lambs analyzed in this work may still be within the BAT to WAT transition period.

As a first step in the interpretation of the transcriptome analysis reported here, we considered it important to determine which genes showed the highest proportions of accumulated gene expression in the fat transcriptome of suckling lambs. The identified CRGs were associated with energy production in the form of ATP in the mitochondrion based on the enrichment analysis performed herein (see the complete list in Supplementary Table S4). This organelle also appeared in some terms in the list (inner mitochondrial membrane protein complex, respiratory chain complex, mitochondrial respirasome, etc.). These results hint at the high metabolic activity occurring in the adipose tissue analyzed in all analyzed samples and agree with the central role that mitochondria play in the metabolism of adipose tissue (59). Moreover, one of the BAT-specific functions is transforming mitochondrial energy into heat in adaptive thermogenesis (59). Our previous study already highlighted this result by comparing the perirenal fat transcriptome of Spanish Assaf and Churra breed suckling lambs (27). From the total of 670 genes identified in that study as CRGs for these two breeds (expression levels >150 FPKM in both breeds), a total of 318 genes (~ 47.5%) are included in the list of Spanish Assaf suckling CRGs reported in this study. The fact that almost half of the genes expressed in the perirenal fat tissue in both studies are common suggests that there is consistency among the fat transcriptomes of suckling lambs, regardless of their breed.

Among the CRGs, focusing on the top 10 most highly expressed genes identified in the two groups of KKCF tissue samples analyzed in this work (Table 1), it was notable that the most highly expressed gene in both the High- and Low-KKCF groups was that encoding fatty-acid-binding protein 4 (*FABP4*; Table 1; Figure 2). *FABP4* is one of the most abundant proteins in mature adipocytes and is considered a genetic marker of WAT (60). However, whether its localization is limited to mature adipocytes is not clear. The expression of *FABP4* has been reported to increase with the progression of adipogenesis in fat tissue beneath the lower dermis in mice (61). *FABP4* physically interacts with *PPARG* (62), which controls the expression of genes responsible for the differentiation of preadipocytes into mature white adipocytes and is considered a key regulator of adipogenesis (63). The expression level of *PPARG*, which is also considered a WAT marker, in the samples analyzed here was also >150 FPKM (Figure 2). Because *FABP4* negatively regulates *PPARG* at the posttranscriptional level and represses preadipocyte differentiation (64), lower expression of *FABP4* has been associated with obesity in different studies (65, 66). In our samples, the expression levels of these two WAT markers, *FABP4* and *PPARG*, were lower in the Low-KKCF group than in the High-KKCF group (see Figure 2), although none of them were identified as DEGs.

Considering the remaining top 10 highly expressed genes in the two groups of samples (Table 1), it seems worth highlighting that many of the genes common to the two lists are related to ribosomes (*RPLP1*, *RPLP2*, *RPL37*, and *RPS2*), most of which are constituents of this organelle. The synthesis of ribosomes and translation are essential for the growth, differentiation, proliferation and development of animal cells (67). A study using human adipose-derived stromal cells (hASCs) reported an overall reduction in protein synthesis after adipogenic induction and a downregulation of the expression and translational efficiency of ribosomal proteins (68). Based on this, the high expression levels of ribosome-related genes identified in both groups of samples under study (High- and Low-KKCF) may indicate that extensive cellular remodeling is taking place in the studied adipose tissue because of the early stage of growth of the suckling lambs analyzed, independent of the proportion of carcass KKCF.

The expression levels of two other important fat tissue gene markers, the *UCP1* and *CIDEA* genes, are also represented in Figure 2. The *UCP1* gene, which is a classical gene marker of BAT, showed very heterogeneous expression levels among samples, and even within groups of samples, there were important differences (209.06 ± 245.06 FPKM). The expression of this gene reached the 150 FPKM threshold only in the Low-KKCF group and not in the High-KKCF group. The identification of gene expression of this BAT marker in some of our suckling lamb samples contrasted with the results reported by Basse et al. (57), who did not observe *UCP1* expression in four-day-old lambs. However, our results agree with those of Suárez-Vega et al. (27) and Yuan et al. (69), who reported expression levels in the perirenal fat adipose tissue of sheep at 17–35 days of age and sheep of 2–12 months of age, respectively. The previously indicated higher expression levels observed for *UCP1* in the Low-KKCF group compared with the High-KKCF group (Figure 2) can be explained by the fact that the Low-KKCF group included animals that were slaughtered at a younger age and could suggest that more thermogenesis was taking place in the adipocytes of the Low-KKCF animals, as *UCP1* represents the key point of this pathway. Indeed, this gene was one of the Low-KKCF DEGs identified in the DE analyses and will be discussed later. On the other hand, the *CIDEA* (cell death-inducing DNA fragmentation factor α-like effector A) gene, which was suggested by Garcia et al. (70) for use as a BeAT marker, as shown in Figure 2, showed consistently high expression levels among samples (226.89 ± 86.73 FPKM) and was included in the list of CRGs reported in this work. This gene is a marker of thermogenesis in adipose tissue and plays an important role in the regulation of this process by inhibiting *UCP1* activity (71, 72). In addition, the CIDEA protein colocalizes around lipid droplets (73). Human *CIDEA* inhibits lipolysis, causing a significant decrease in glycerol release (74). Because *CIDEA* expression occurs specifically in BAT and BeAT, this is an interesting marker gene for performing further studies on its role in the development of adipose tissue.

The experimental design of this study gave us the opportunity to identify a set of 80 genes whose expression levels may influence fat deposition levels in perirenal and visceral depots in Spanish Assaf suckling lambs. We acknowledge that the number of samples analyzed in the present study was limited, although it was higher than the minimum number of biological replicates recommended by Conesa et al. (75) for RNA-Seq studies and similar to the number of samples analyzed in other recent studies evaluating the fat transcriptome in sheep (30, 35, 76). However, an additional limitation to our experimental design in this specific study was the significant differences in age in the two groups of animals initially considered based on their different levels of perirenal fat accumulation. Although these differences supported the general observation that the lambs slaughtered at an older age show more fat accumulation than younger lambs, individual deviations from this general observation are of special interest when studying the “fatness” phenotype. Hence, the presence of the two animals in the study that did not conform to the fatness-age correlation (one animal was Low-KKCF and Old, and one other animal was High-KKCF and Young) led us to include the “age” category in our model. Although the contribution of the inclusion of this second category in our DE model may appear limited due its nonuniform distribution in the two considered fatness groups (with only 2 animals that did not show the fatness-age correlation), we consider these animals to have provided valuable information that helped us to identify a set of DEGs and enriched terms that were specifically associated with fatness levels in the KKCF depots of suckling lambs, independent of the time needed to reach the slaughter weight defined by market criteria.

In relation to the DEGs identified in the two groups defined based on fatness accumulation in KKCF depots in this study, the results of the enrichment analyses (graphically summarized in Figures 3, 4) highlight relevant metabolic and developmental processes that clearly differentiate the two groups. Considering the LowKKCF-DEGs, the significant enriched terms were globally summarized as being related to fatty acid metabolic processes and other metabolic pathways associated with obtaining ADP and ATP (“*purine nucleoside and ribonucleoside biphosphate metabolic process” and “ribonucleoside and nucleoside biphosphate and phosphate metabolic process”*), whereas for the High-KKCF group, the enriched terms were globally grouped with terms related to vessel endothelial development (regulation of blood vessel cell endothelial cell proliferation, fibroblast migration and regulation) and cellular response mechanisms toward acid chemicals (e.g., “*response to amino acid*,” “*cellular response to amino acids*” among others). Below, we present a discussion of these results, attempting to explain their biological meaning in relation to the analysis performed.

Focusing first on the Low-KKCF-related results, the genes supporting most of the enriched terms identified in this group were directly related to lipid metabolism and different metabolic processes related to nucleoside and ribonucleoside (especially purine) bisphosphates (Supplementary Table S6). Some of these genes were *ACSS2*, *ACLY*, *ELOVL6*, *DGAT2*, *FASN* and *GPAM*. Among the lipid metabolism-related terms, we found a large variety of processes, such as “*fatty acid biosynthetic process*” and “*cholesterol biosynthetic process*.” These significantly enriched pathways suggest that the synthesis of many types of lipids occurs in the adipose tissue of Low-KKCF animals. For instance, the *FASN* gene encodes fatty acid synthase, which is a central enzyme in *de novo* lipogenesis. FASN is an enzyme that catalyzes a rate-limiting step in *de novo* fatty acid synthesis in ruminants, as its main function is to catalyze the synthesis of long-chain saturated fatty acids (77). In addition, the increased expression in the Low-KKCF group of genes such as *DGAT2* and *GPAM,* which encode key enzymes for the synthesis of triacylglycerols (78, 79), also suggests that in the perirenal depots of these animals, the biosynthesis of these compounds also takes place. The mentioned synthesis processes are linked with *ELOVL6*, a gene that supports a remarkably large number of significantly enriched terms identified in the Low-KKCF group (see Supplementary Table S6). This gene is considered a key gene involved in regulating the balance of the fatty acid composition, as it is involved in the elongation of long-chain saturated and monounsaturated fatty acids (80–82). The identification of *ELOVL6* as a LowKKCF-DEG highlights the potential importance of remodeling processes, in addition to fatty acid synthesis, in the perirenal adipose tissue of these animals compared with the High-KKCF group.

Considering these results and a detailed review of gene expression and transcriptomic studies focusing on fat deposition in livestock species, such as those comparing animals with higher and lower fatness levels, we can see that the expression levels of all the mentioned genes are generally associated with the groups of animals showing higher fat deposition levels. This has been shown in relation to different fat depots in pigs (29, 83), calves (84), and sheep (35) and suggests that these genes are general genes that are components of the universal genetic basis of fat deposition. Hence, we acknowledge that the fact that all the mentioned genes related to biosynthesis and lipid remodeling showed higher expression levels in the animals with lower KKCF values in our study appears to be a discrepancy. However, as previously stated, for the interpretation of our results, it is important to consider the critical growth stage of the suckling lambs analyzed in the present study, which were slaughtered at a very early age (17–27 days for the Low-KKCF group), resulting in a completely different situation than that of the animals analyzed in the previously mentioned studies on livestock fat deposition, in which fat tissue development had already been completed. Indeed, as previously noted, the lambs analyzed here may still be within the BAT to WAT transition period (based on Figure 2).

In this regard, it is interesting that the role of *ELOVL6* in the regulation of fatty acid length is important for BAT and BeAT function (85). In mouse adipose tissue, the loss of the *ELOVL6* gene causes reduced expression of components of the mitochondrial electron transport chain, which leads to a reduction in the thermogenic capacity (86). Indeed, this gene, together with *UCP1, SCD*, *LEP* and *PPARGC1B*, contributed to the enrichment of terms such as “*positive regulation of cold-induced thermogenesis*,” “*cold-induced thermogenesis*,” and “*adaptive thermogenesis*” among the LowKKCF-DEGs (Supplementary Table S6). The identification of these enriched terms may help to explain the previously mentioned discrepancy of higher lipid biosynthesis in the Low-KKCF group compared with the High-KKCF group because at least some of the fatty molecules generated in these biosynthesis processes could be used for such thermogenic activity. The increased expression of the *UCP1* gene in the Low-KKCF group also contributed to the enrichment of the “*mitochondrial inner membrane*” term, which is directly related to UCP1 because this protein is inserted in this membrane. Additionally, *UCP1* was involved in the enrichment of terms such as “*fat cell differentiation*,” “*brown fat cell differentiation*,” “*response to dietary excess*,” “*response to nutrient levels*,” and “*response to fatty acid levels*,” among others (Supplementary Table S6). The enrichment of these terms was supported by other genes, including *LEP* or *DGAT2* in many cases.

The *LEP* gene encodes leptin, an adipokine produced by adipose tissue as a feedback mechanism that leads to appetite reduction and has been shown to inhibit glucose conversion to lipids and fatty acid incorporation by porcine adipocytes (87). In livestock studies focused on adult animals higher expression levels of this gene are normally associated with animals with higher fat deposition levels (29, 88). However, in young animals, leptin has been shown to affect growth, and periods of rapid growth require high levels of LEP (89–91). Hence, different studies have suggested that leptin acts as a skeletal growth factor and an inducer of longitudinal growth (92, 93). All of these findings and inferences are in accord with the identification of increased expression of *LEP* in Low-KKCF animals, which in general reached the commercial slaughter weight of 11–12 kg at a younger age. Additionally, our results agree with those of Xing et al. (94), who compared the Landrace breed of pigs, characterized by high growth rates and lean carcass percentages, with the Songliao breed, with higher fat deposition. In this case, the Landrace breed showed the highest levels of *LEP* expression.

Finally, in relation to the previously mentioned increase in *UCP1* expression observed in the Low-KKCF group, we should take into account that while free fatty acids activate UCP1, purine nucleotides, such as ATP, strongly inhibit the function of UCP1 (95). In this context, we also found enriched terms for the LowKKCF-DEGs associated with purine nucleotide/ribonucleotide metabolic processes (Figure 3A), supported by genes such as *ACSS* and *ACLY,* which are both related to acetyl-CoA metabolism (96). Hence, although our results suggested that the Low-KKCF animals were at an earlier stage of adipose tissue development than the High-KKCF animals, where BAT-related processes such as thermogenesis were still observed, the potential inhibition of the UCP1 function identified in the Low-KKCF group suggested that they may have been in the last stages of the BAT to WAT transition. This was also supported by the overall higher expression of WAT gene markers than BAT gene markers shown for both groups in Figure 2.

Focusing now on the enrichment results obtained for the HighKKCF-DEGs (Figure 4; Supplementary Table S7), we found several terms related to blood vessel endothelial cell proliferation connected to terms associated with the cellular response to acid chemicals through a central gene, *VEGFA.* This gene is a member of the PDGF/VEGF growth factor family and encodes vascular endothelial growth factor A (VEGFA), which induces the proliferation and migration of vascular endothelial cells and is essential for both physiological and pathological angiogenesis (97). VEFG-A is also a crucial molecule controlling vasculogenesis, vascular permeability and tissue remodeling (98). Angiogenesis, the process of forming new blood vessels, is crucial to adipogenesis (99), and VEGF-A is responsible for most of the proangiogenic activity in fat tissue (100, 101). Since the vasculature is essential for adipose tissue expansion, as it supplies oxygen and nutrients and is also responsible for the transport of fatty acids to other tissues, angiogenesis is considered to be a rate-limiting step in fat tissue expansion (102). VEGF-A levels are regulated by both external factors, such as exercise (103) and dietary protein levels (104), and internal factors, such as hypoxia and various growth factors (105, 106). Adipocytes store lipids in lipid droplets when nutrients are abundant, and as they increase in size, oxygen becomes less available, which leads to a hypoxic status. In this context, hypoxia has been described as the most important mechanism for VEGF modulation (107). This would explain the connection of *VEFG* with the enriched terms “*response to acid chemical*” and “*cellular response to acid chemical*.” All of these findings and inferences agree with the observed increase in *VEGF-A* gene expression levels in the High-KKCF group compared with the Low-KKCF animals, as their larger fat depots could have reached the condition of moderate hypoxia with the subsequent activation of the angiogenesis process in response (108). In relation to angiogenesis, *VEGF-A* is connected to terms related to fibroblast migration through the *THBS1* gene. Thrombospondin 1 (THBS1) is a large adhesive extracellular matrix glycoprotein (109) that is expressed predominantly in visceral adipose tissues, and its expression has been found to be elevated in obese humans (110). According to Kong et al. (111), *THBS1* overexpression promotes adipose tissue development and weight gain in mice. This biological role agrees with our results, as *THBS1* was one of the HighKKCF-DEGs identified in our analysis. Interestingly, neither *VEGF-A* nor *THBS1* have been reported as DEGs in general studies comparing adult animals with high and low fatness levels. This suggests that the vasculogenesis process associated with these genes may be a critical stage of fat tissue development in young animals, such as the suckling lambs analyzed here.

Other enriched terms found for the HighKKCF-DEGs related to acid chemical responses were “*cellular response to amino acid stimulus*,” “*response to leucine*,” “*response to leucine starvation*,” “*cellular response to leucine*” and *“cellular response to leucine starvation*” (Supplementary Table S7). The most relevant genes supporting these terms, *SENS1* and *SESN2*, encode sestrins. Sestrins are a family of stress-inducible proteins that regulate metabolism based on sensing nutrient levels and redox status in cells that participate in the negative regulation of mammalian target of rapamycin complex 1 (mTORC1). Several terms related to mTOR were also enriched in the analysis of the HighKKCF-DEGs (“*TOR signaling,” “negative regulation of TORC1 signaling,” “regulation of TORC1 signaling,” “negative regulation of TOR signaling”*; Supplementary Table S7). mTOR is a complex that also plays important roles in adipocyte development and functional maintenance (112), for which a positive role in adipogenesis has been classically described (113). In the High-KKCF animals, increased levels of sestrin expression, leading to negative regulation of mTOR signaling (as suggested by the identified enriched terms previously mentioned), would be associated with the inhibition of adipogenesis. This would indicate that the developmental stage of the perirenal fat of these animals had moved beyond the preadipocyte differentiation phase toward an expansion phase, for which vasculogenesis is essential. In turn, the lower expression of sesrins in the Low-KKCF group would suggest that adipogenesis is activated in this group.

Overall, the transcriptomic analysis of perirenal fat in suckling lambs presented herein suggests that a higher content of KKCF in the carcass of suckling lambs is associated with a more mature stage of perirenal adipose tissue, associated with increased vasculogenesis and expansion of the tissue, where thermogenesis and adipogenesis are inhibited, compared with the Low-KKCF group. We have identified genes such as *VEGF-A*, *THBS*, *SESN1* and *SESN2* as being associated with this more advanced developmental stage of perirenal fat depots with higher KKCF values, which are associated with better carcass quality. While we acknowledge that the limited number of samples analyzed means that these results should be confirmed by future studies involving large population sizes, the findings presented here represent a valuable first attempt to understand the molecular mechanisms underlying fat deposition in suckling lambs. In addition, the genes indicated above are proposed as candidate genes, and their polymorphisms should be evaluated for potential direct associations with carcass fatness traits in suckling lambs.

## Data availability statement

The data presented in the study are deposited in the BioStudies repository, accession number E-MTAB-11839, and can be found at: https://www.ebi.ac.uk/biostudies/arrayexpress/studies/E-MTAB-11839.

## Ethics statement

Ethical review and approval was not required for the animal study because the lambs included in this experiment were not subjected to any experiment, and their management was carried out following the usual management practices on farms raising suckling lambs with artificial lactation. The management, transport, and slaughter of the animals at a local slaughterhouse were in accordance with Spanish and EU legislation [Spanish Laws 32/2007, 6/2013, and RD 37/2014; Council Regulation (EC) 199/2009].

## Author contributions

MA-G: formal analysis and writing—original draft. AS-V: conceptualization, validation, and writing—review and editing. PF, RP, and HM: investigation. JM: methodology and investigation. J-JA: conceptualization and investigation. BG-G: conceptualization, methodology, and writing—review and editing. All authors contributed to the article and approved the submitted version.

## Funding

The research described here has been funded by the project EpiMilksheep (RTI2018-093535-B-100) funded by the Spanish Ministry of Science and Innovation. MA-G is funded by a predoctoral fellowship from the Junta de Castilla and León Government and the European Social Fund.

## Conflict of interest

The authors declare that the research was conducted in the absence of any commercial or financial relationships that could be construed as a potential conflict of interest.

## Publisher’s note

All claims expressed in this article are solely those of the authors and do not necessarily represent those of their affiliated organizations, or those of the publisher, the editors and the reviewers. Any product that may be evaluated in this article, or claim that may be made by its manufacturer, is not guaranteed or endorsed by the publisher.

## Footnotes

^1^ https://www.ebi.ac.uk/biostudies/
