# Supplementary material for: Transcriptome analysis of perirenal fat from Spanish Assaf suckling lamb carcasses showing different levels of kidney knob and channel fat
Source: Front Vet Sci. 2023 May 15;10:1150996. doi: 10.3389/fvets.2023.1150996 (PMC10225515; doi:10.3389/fvets.2023.1150996)
Supplement: Supplementary file 9 [file Data_Sheet_1.DOCX]

Supplementary Material

Transcriptome analysis of the perirenal fat from Spanish Assaf suckling lamb carcasses showing different levels of kidney knob and channel fat

María Alonso-García, Aroa Suárez-Vega, Pablo A. S. Fonseca, Héctor Marina, Rocío Pelayo, Javier Mateo, Juan-José Arranz, Beatriz Gutiérrez-Gil^*^

*** Correspondence:** Corresponding Author: beatriz.gutierrez@unileon.es

# Supplementary Figures and Tables

## Supplementary Figures


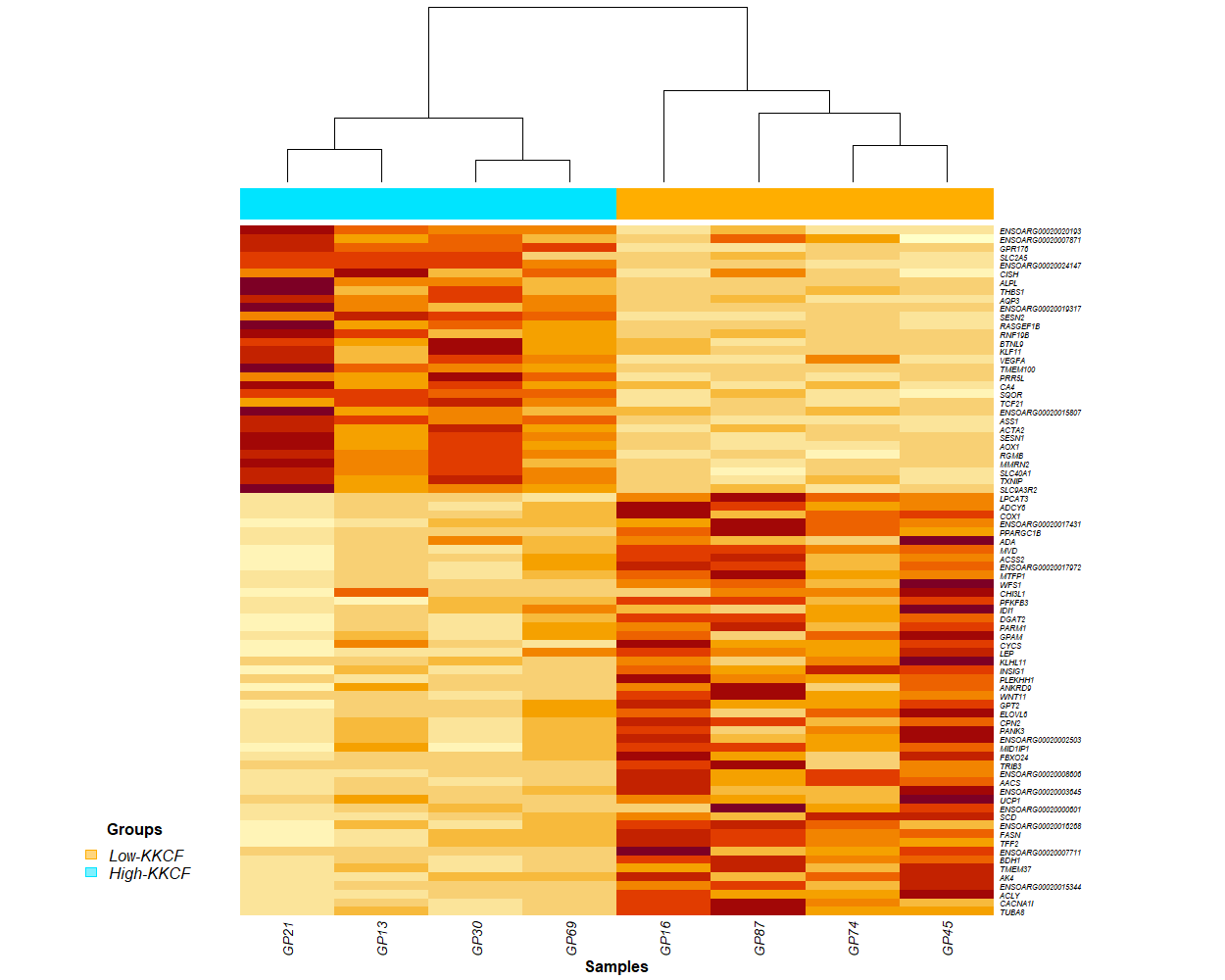


**Supplementary Figure 1.** Heatmap showing the expression level of the 80 differentially expressed genes (DEGs) identified in the comparison between the two groups of suckling lamb perirenal fat transcriptomes contrasted. The dendrogram at the top of the plot shows the clustering of the analyzed samples, which agrees with the two groups contrasted: High-KKCF lambs (blue) and Low-KKCF lambs (orange). In the heatmap, darker colors indicate a higher expression in normalized counts.

**
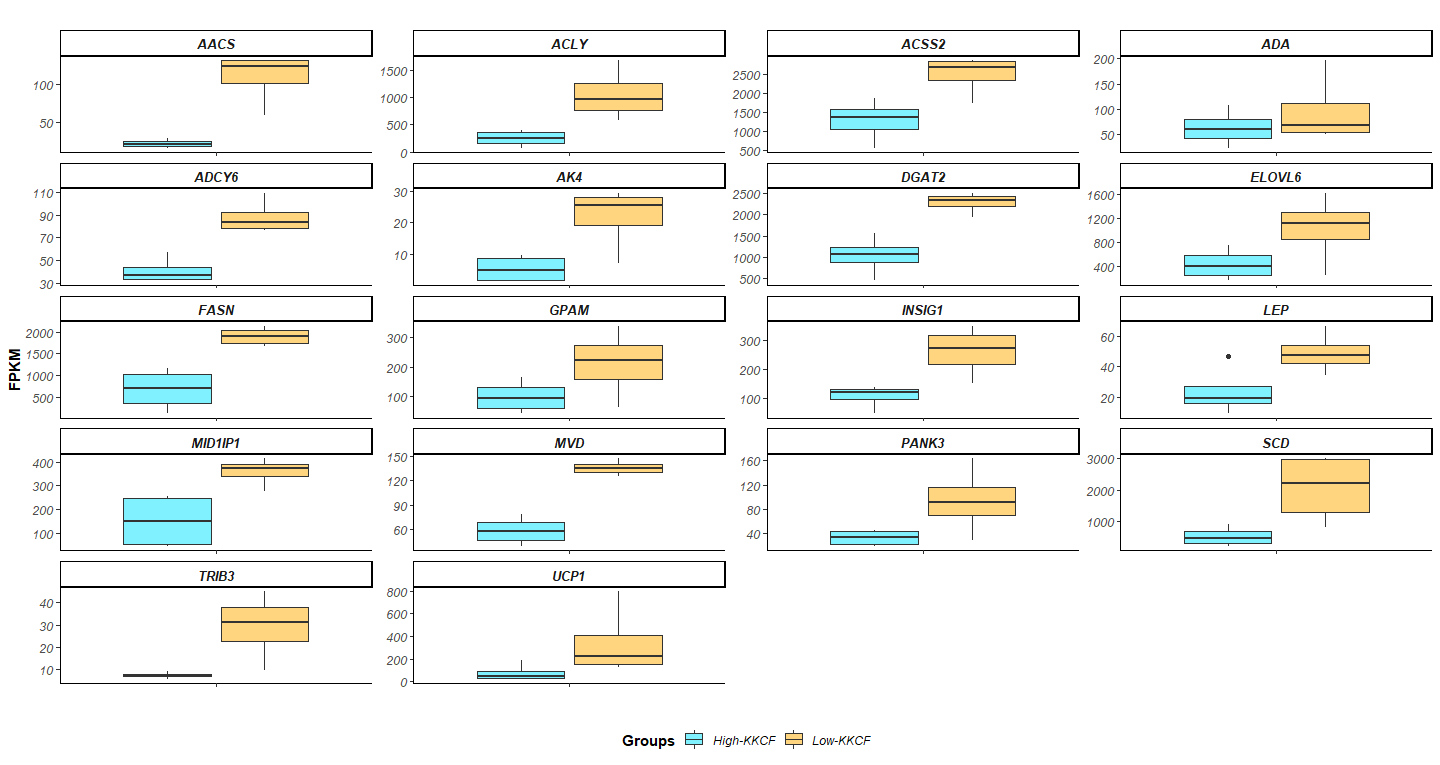
**

**Supplementary Figure 2.** Box plot comparing the mean gene expression levels (FPKM) of each gene between the two groups of samples considered, High-KKCF (blue) and Low-KKCF samples (orange). Graphical representation of the expression level of the differentially expressed genes showing higher expression in the Low-KKCF samples (LowKKCF-DEGs) highlighted by the corresponding enrichment analysis shown in **Figure 3**.

**
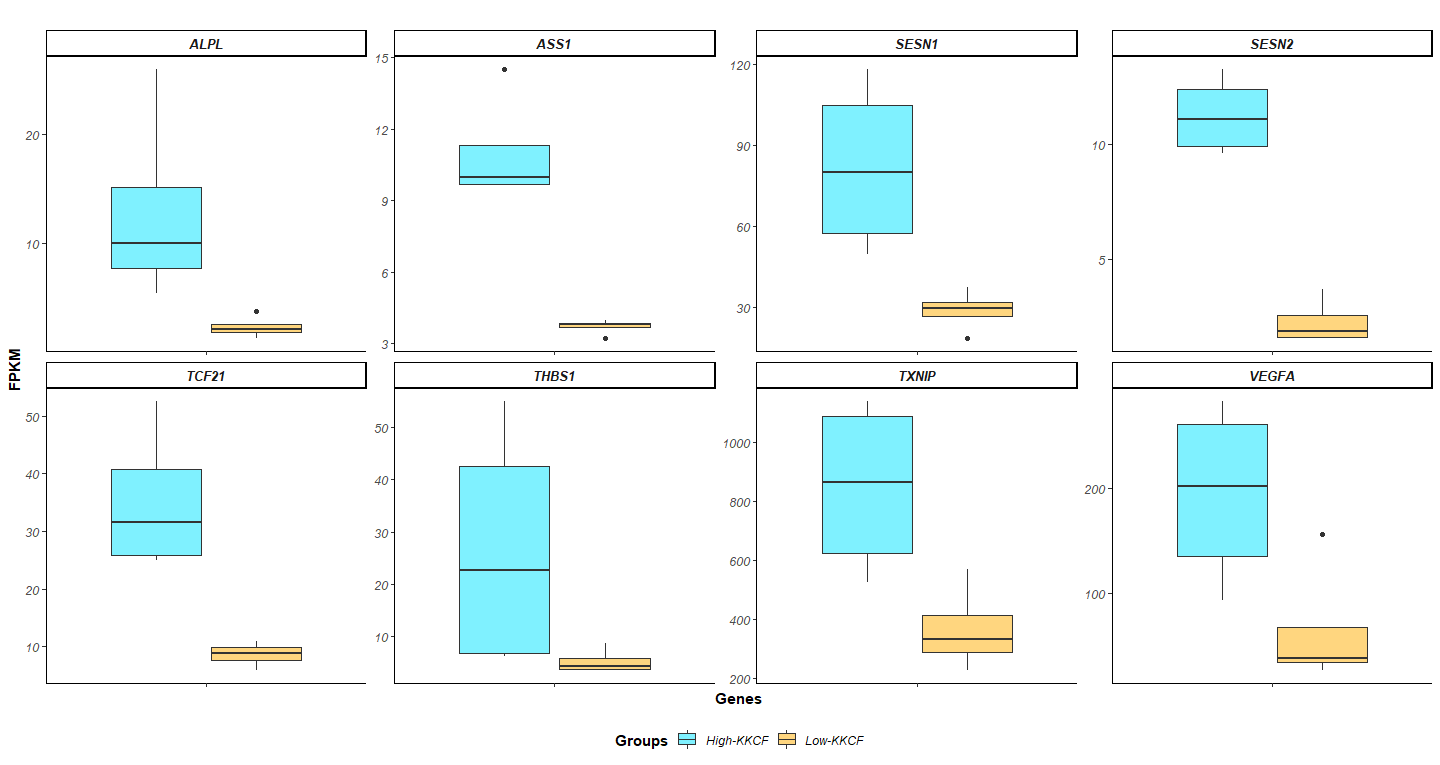
Supplementary Figure 3**. Box plot comparing the mean gene expression levels (FPKM) of each gene between the two groups of samples considered, High-KKCF (blue) and Low-KKCF samples (orange). Graphical representation of the expression level of the differentially expressed genes showing higher expression in the High-KKCF samples (HighKKCF-DEGs) highlighted by the corresponding enrichment analysis shown in **Figure 4**.
